# Partial Regulatory T Cell Depletion Prior to Acute Feline Immunodeficiency Virus Infection Does Not Alter Disease Pathogenesis

**DOI:** 10.1371/journal.pone.0017183

**Published:** 2011-02-25

**Authors:** S. Rochelle Mikkelsen, Julie M. Long, Lin Zhang, Erin R. Galemore, Sue VandeWoude, Gregg A. Dean

**Affiliations:** 1 Center for Comparative Medicine and Translational Research, College of Veterinary Medicine, North Carolina State University, Raleigh, North Carolina, United States of America; 2 Department of Microbiology, Immunology, and Pathology, College of Veterinary Medicine and Biomedical Sciences, Colorado State University, Fort Collins, Colorado, United States of America; Institut Pasteur, France

## Abstract

Feline immunodeficiency virus (FIV) infection in cats follows a disease course similar to HIV-1, including a short acute phase characterized by high viremia, and a prolonged asymptomatic phase characterized by low viremia and generalized immune dysfunction. CD4^+^CD25^hi^FoxP3^+^ immunosuppressive regulatory T (Treg) cells have been implicated as a possible cause of immune dysfunction during FIV and HIV-1 infection, as they are capable of modulating virus-specific and inflammatory immune responses. Additionally, the immunosuppressive capacity of feline Treg cells has been shown to be increased during FIV infection. We have previously shown that transient in vivo Treg cell depletion during asymptomatic FIV infection reveals FIV-specific immune responses suppressed by Treg cells. In this study, we sought to determine the immunological influence of Treg cells during acute FIV infection. We asked whether Treg cell depletion prior to infection with the highly pathogenic molecular clone FIV-C36 in cats could alter FIV pathogenesis. We report here that partial Treg cell depletion prior to FIV infection does not significantly change provirus, viremia, or CD4^+^ T cell levels in blood and lymphoid tissues during the acute phase of disease. The effects of anti-CD25 mAb treatment are truncated in cats acutely infected with FIV-C36 as compared to chronically infected cats or FIV-naïve cats, as Treg cell levels were heightened in all treatment groups included in the study within two weeks post-FIV infection. Our findings suggest that the influence of Treg cell suppression during FIV pathogenesis is most prominent after Treg cells are activated in the environment of established FIV infection.

## Introduction

The acute phase of infection with HIV-1, simian immunodeficiency virus (SIV), and feline immunodeficiency virus (FIV) is characterized by robust viral replication that is usually controlled, but not eliminated, by innate, cell-mediated, and humoral antiviral immune responses [1], [2], [3], [4]. During this acute phase, Treg cells have been variably reported to increase, decrease or not change depending on the virus/host system and tissues evaluated. An important question is whether Treg cells, present or induced at the time of infection, suppress a robust anti-viral immune response. This question is complicated by data that show Treg cells are susceptible to lentiviral infection and are depleted along with conventional CD4^+^ T cells during acute lentiviral infection [5], [6], [7]. The clinically relevant issue is whether depletion of Treg cells during the acute phase of HIV-1 infection might alter the viral and/or immunological set-points thereby improving clinical outcome.

In vivo depletion of Treg cells is complicated by the fact that no Treg specific cell surface marker has been identified. The IL-2 receptor alpha chain, CD25, remains the best target although CD25 is neither universally expressed on cells with regulatory function nor is it specific since many activated cell types are known to express CD25. Nevertheless, in vivo depletion of CD25^+^ Treg cells is under intense investigation as a primary or adjunctive immunotherapy against various types of cancer [8]. If Treg cell depletion was to be attempted in acutely HIV-1 infected people, drugs that target CD25 would be the logical choice.

Feline immunodeficiency virus is a natural lentiviral pathogen of outbred domestic and wild cats that causes an immunodeficiency syndrome very similar to HIV/AIDS. It has previously been shown that feline Treg cells are activated and more suppressive during the acute and chronic phases of FIV infection [9], [10]. Similarly, HIV-1 can enhance human Treg cell suppressive capacity [11], [12]. Multiple studies have quite clearly shown that Treg cells from hosts infected with HIV-1, SIV or FIV suppress antiviral responses during the chronic phase of disease [5], [13], [14], [15], [16], [17]. We have previously shown that transient in vivo Treg cell depletion during chronic FIV infection unmasks FIV-specific immune responses [18]. Only a few studies have addressed this issue in the acute phase. A correlation between Treg cell induction and limited anti-SIV immune responses during acute infection has been demonstrated [19] and individuals with more robust anti-lentiviral responses possess lower Treg cell frequencies [5], [7], [20]. These data suggest that Treg cell suppression of antiviral immunity may occur at the earliest stages of the immune response.

In this study we sought to determine whether Treg cell depletion using an anti-feline CD25 monoclonal antibody prior to natural lentiviral infection in the well-characterized FIV/cat model would alter the immunologic or virologic set-point. We hypothesized that this could occur either due to removal of Treg cell immunosuppression leading to heightened antiviral responses and/or due to removal of Treg cells that host lentiviral infection and replication. We report here that Treg cell depletion prior to FIV infection does not significantly change viral load or CD4^+^ T cell levels in tissues. The effects of anti-CD25 mAb treatment are truncated in cats acutely infected with FIV as compared to chronically infected cats or FIV naïve cats, as Treg cell levels were heightened in all treatment groups within two weeks following FIV infection. We propose that the influence of Treg cell suppression during FIV infection is most prominent after established infection, when Treg cells are activated and more functionally suppressive.

## Materials and Methods

### Ethics Statement

All experimental manipulations and protocols (08-019-B) were approved by the North Carolina State University Institutional Animal Care and Use Committee. The animals were housed and maintained in accordance with standards established in the Animal Welfare Act and the Guide for the Care and Use of Laboratory Animals.

### Animals, Viral Inoculum, and Monoclonal Antibody Administration

A total of 24 female specific-pathogen-free (SPF) cats were purchased from Liberty Labs (Liberty, NY). Mouse anti-feline CD25 hybridoma (9F23) [21], a generous gift from M. Honda of the National Institutes of Health in Tokyo, Japan, and mouse anti-yellow fever antigen (YFA) hybridoma (CRL-1689; ATCC, Manassas, VA) were grown in serum-free medium. Antibody was purified and certified endotoxin- and mycoplasma-free by Leinco, Inc. (St. Louis, MO). One group of cats received a single injection of 9 mg/kg anti-feline CD25 mAb intraperitoneally (i.p.) between 25 and 31 weeks of age and 12 days prior to infection with FIV-C36 [22]. Another group was treated with 9 mg/kg anti-YFA mAb i.p. as a mouse IgG2a κ light chain isotype control mAb and a third group was treated with 3 mL/kg 0.9% sodium chloride (Hospira, Lake Forest, IL) i.p. as a vehicle control. All cats were infected with 1×10^5.2^ TCID_50_ units molecular clone FIV-C36 [23] intravaginally along with 1×10^5.2^ TCID_50_ units FIV-C36 intravenously 12 days after mAb or saline treatment. Titered FIV-C36 was a generous gift from Dr. Sue VandeWoude of Colorado State University.

### Sample Collection and Processing

Whole blood was collected into Vacutainer tubes (BD, Franklin Lakes, NJ) containing ACD on days −12, 0, 7, 14, 35, and 54 post-infection (p.i.). Peripheral blood mononuclear cells (PBMCs) were isolated by centrifugation of blood over Histopaque (Sigma, St. Louis, MO) and serum was processed as previously described [24], [25]. Blood for plasma isolation, complete blood counts, and leukocyte differentials was collected in Vacutainer tubes containing EDTA. Plasma was isolated by centrifugation (1100×*g* for 10 min) and aliquots were frozen at −80°C for subsequent RNA isolation. Popliteal lymph node (LN) biopsies were performed on anesthetized cats on days 14 and 35 p.i. At day 54 p.i., mesenteric and retropharyngeal LNs in addition to spleen were harvested and processed as described previously [26]. Cells were used immediately in ex vivo assays or cell aliquots were frozen at −80°C for subsequent DNA isolation.

### Phenotypic Analysis

At least 1×10^6^ cells were labeled with the following antibodies for flow cytometric analysis. Antibodies against feline CD3 (NZM1) [27], CD4 (30A) [28], CD8α (3.357) [28], and IFN-γ (E6D4A5) [29] were purified from hybridoma supernatants in our lab. Anti-CD80 (B7.1.66) was generously provided by Dr. Mary Tompkins of North Carolina State University [30]. Anti-CD1a (Fe1.5F4) was provided by Dr. Peter Moore of the University of California at Davis [31]. Antibodies against CD56 and IL-2 were purchased from BioLegend (San Diego, CA). Antibodies against Ki67 and TNF-α were purchased from BD Biosciences (San Diego, CA). Anti-B220 was purchased from Southern Biotech (Birmingham, AL). Anti-TGF-β was purchased from R&D Systems (Minneapolis, MN). Anti-MHC II was purchased from Serotec (Raleigh, NC). Some antibodies were conjugated to Pacific Blue, Pacific Orange, Alexa Fluor 647, or biotin using kits from Invitrogen Molecular Probes (Carlsbad, CA). Some antibodies were conjugated to PerCP or PE using kits from Prozyme (San Leandro, CA). Mouse anti-feline CD25 (9F23) was conjugated to FITC using standard protocols. Anti-mouse IgG3-APC (Jackson ImmunoResearch, West Grove, PA), anti-mouse IgG3-APC-Cy7 (Southern Biotech), and streptavidin-Pacific Orange (Invitrogen) were used for secondary detection. Intracellular FoxP3 staining was performed with eBioscience FoxP3 staining buffers and FoxP3-PE-Cy7 (FJK-16s; San Diego, CA) according to manufacturer's protocol, with the exception that cells remain in the permeabilization/wash buffer no longer than 30 min. For intracellular cytokine staining, cells were incubated with 1× monensin (BioLegend, San Diego, CA) for six hr, labeled for surface markers, fixed with 4% paraformaldehyde, permeabilized with BD Cytofix/Cytoperm kit Perm/Wash buffer, and stained with anti-cytokine mAbs. For intracellular Ki67 staining, cells were first labeled for surface markers, incubated with BD Cytofix/Cytoperm for 15 min, washed with BD Perm/Wash buffer, incubated with BD Cytoperm Plus for 10 min, washed, and incubated with BD Cytofix/Cytoperm for 5 min. Cells were then labeled with anti-Ki67. Flow cytometric analysis was performed using an LSR II flow cytometer and FacsDIVA software (BD). 100,000–1,000,000 gated events were collected per sample.

### Viral Parameters

Quantitative real-time one-step reverse transcriptase (RT)-PCR assays to quantify viremia were performed on a Bio-Rad MyiQ™ PCR detection system (Hercules, CA). RNA was extracted from plasma using the QIAamp viral RNA mini kit (QIAGEN, Valencia, CA) following manufacturer's protocol. Detection of plasma viremia in RNA samples was performed using FIV-C *gag* specific primers 704f and 756r and probe 727p [32]. Each RNA sample was combined with TaqMan RT-PCR Mix (Applied Biosystems, Branchburg, NJ), TaqMan RT Enzyme Mix (Applied Biosystems), 400 nM forward and reverse primers, and 80 nM TaqMan probe. Plasma viremia RT-PCR cycling conditions were as follows: 30 min at 48°C, 10 min at 95°C, and 50 cycles of a 15 sec step at 95°C followed by a 1 min step at 55.5°C. Fluorescence was recorded at the end of each annealing/extension step. RNA standards were generated by in vitro transcription of a pGEM-T Easy plasmid (Promega, Madison, WI) encoding FIV clade C *gag* using the T7 MAXIscript kit (Applied Biosystems) according to manufacturer's instructions. A 10-fold dilution series of RNA standards provided a detection range from 10^1^ to 10^5^ RNA molecules per reaction. Bio-Rad MyiQ™ optical system software v2.0 was used to generate a standard curve; viral RNA copies/mL plasma was subsequently calculated.

To detect proviral load in PBMC and tissue samples, quantitative PCR was performed on DNA samples extracted with the DNeasy blood and tissue kit (QIAGEN). Each amplification reaction included 0.5 µg DNA sample, TaqMan universal PCR master mix (Applied Biosystems), 400 nM 704f and 756r primers, and 40 nM 727p probe. Real-time PCR cycling conditions were as follows: 2 min at 50°C, 10 min at 95°C, and 45 cycles of a 15 s step at 95°C followed by a 1 min step at 55.5°C. To normalize proviral load per 10^6^ cells, a 10-fold dilution series of a pGEM-T Easy plasmid encoding FIV C *gag* was used to provide a detection range of 10^1^ to 10^5^ copies. A quantitative PCR was performed to detect CCR5 copies in 0.5 µg DNA sample as previously described [18]. The limit of detection was ≤10 copies of FIV per 1 µg DNA. Standards, controls, and samples were run in duplicate for real-time PCRs.

### Peptides

The entire FIV p24 protein sequence was synthesized by JPT (Springfield, VA) consisting of 40 15-aa peptides overlapping by 10 aa. Peptides were reconstituted at 1 mg/mL in 1× PBS, 10% dimethyl sulfoxide (DMSO), or 100% DMSO according to solubility and stored at −80°C.

### Interferon-γ ELISpot

Capture and detection antibodies from the feline IFN-γ detection module (R&D Systems) were used with ELISpot Immobilon-P 96-well plates (Millipore, Bedford, MA) to quantify IFN-γ-producing cells after stimulation with FIV p24 peptides as previously described [33]. The protocol was modified to use of 2.5 or 5×10^5^ fresh instead of frozen cells/well.

### IgG Titers Determined by ELISA

Serum IgG titers against mouse antibody were determined by ELISA as previously described [22]. For the FIV p24 antibody ELISA, Immulon-2HB plates (Nalge Nunc International, Rochester, NY) were coated with 1.0 µg/mL p24-GST fusion protein and the assay was performed as previously described [34]. For the FIV gp95 antibody ELISA, Lumitrac 600 high-binding plates (Greiner Bio-One, Monroe, NC) were coated with 2.5 µg/mL gp95-Fc antigen [35] diluted in carbonate buffer (15 mM Na_2_CO_3_, 35 mM NaHCO_3_, pH 9.5). Plates were blocked with 200 µL/well blocking buffer (3% nonfat dry milk, 5% goat serum, 0.1% Kathon in carbonate buffer) for 2 hr at room temperature. Plates were washed four times with wash buffer (0.5% Tween 20 in 1× PBS). Serum samples were diluted in complete sample diluent (2.5% human serum, 1% bovine serum albumin, 1% nonfat dry milk, 0.05% Tween 20, 0.1% Kathon in 1× PBS) and added to blocked plates at 100 µL/well. Plates were incubated for three hr at 37°C and then washed four times with wash buffer. Antibody was identified with goat anti-cat IgG-horseradish peroxidase (HRP) (Bethyl Labs, Montgomery, TX) diluted 1∶80,000 in complete sample diluent and incubated for one hr at room temperature. Plates were washed five times. 100 µL/well Pierce SuperSignal ELISA Femto chemiluminescent substrate (Rockford, IL) was added to plates. Plates were read on a Perkin Elmer Wallac Victor^3^ 1420 Multilabel Counter (Waltham, MA) 2 min after addition of substrate. A threefold higher optical density of tested sample over pre-infection samples determined positive antibody titers.

### Statistics

Comparisons between post-treatment or post-infection data to baseline values within groups were made using unpaired *t*-tests. Comparisons between treatment groups were made using 1-way ANOVA with Tukey's post-test. Statistics were calculated using GraphPad Prism version 5.0 (GraphPad Software, San Diego, CA).

## Results

### Anti-CD25 monoclonal antibody treatment depletes Treg cells in cats prior to FIV infection

**Figure 1:**
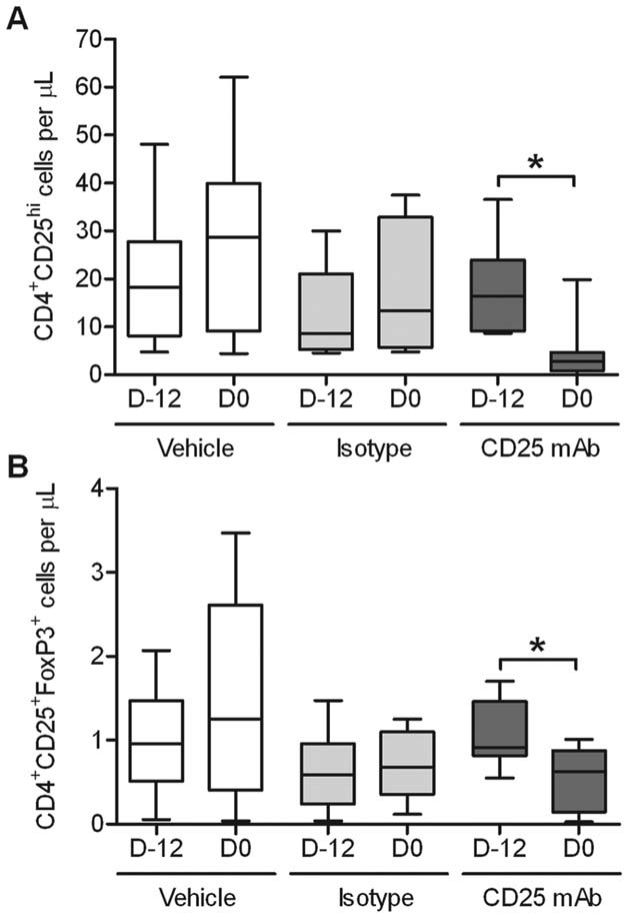
Anti-CD25 monoclonal antibody treatment depletes Treg cells in cats. Cats received 9 mg/kg anti-CD25 mAb (9F23) (n = 8) or 9 mg/kg isotype control mAb (CRL-1689) (n = 8) i.p. on day −12. A third group of cats received 3 mL/kg saline on day −12 (n = 8). (A) PBMCs were labeled with antibodies for CD4 and CD25 and analyzed by flow cytometry for CD4^+^CD25^hi^ percentages within the lymphocyte population on days −12, 0, 7, 14, 35, and 54 post-FIV infection. Percentages were multiplied by lymphocyte absolute number to quantify absolute numbers of CD4^+^CD25^hi^ cells. (B) CD4^+^CD25^+^FoxP3^+^ cell percentages were determined by flow cytometry. CD4^+^CD25^+^FoxP3^+^ absolute number was calculated. Mean ± SEM is shown. * indicates *p*<0.05.

Twenty-four SPF cats were divided into three groups of eight cats each, with siblings distributed across the groups. Cats received anti-CD25 mAb, isotype control mAb, or saline by i.p. route on day −12 relative to the date of infection with FIV-C36. FIV-C36 is a highly pathogenic molecular clone of an FIV clade C isolate [23]. Acute FIV-C36 infection is characterized by very high peak viremia and substantial CD4^+^ T cell loss, leading to early symptoms of immunodeficiency [23], [36]. Cats received 2×10^5.2^ TCID_50_ units FIV-C36 on day 0, at the previously determined nadir of CD4^+^CD25^hi^ cell depletion following treatment with anti-CD25 mAb [18], [22]. Expression of CD25 and FoxP3 were used to identify Treg cells. Treg cells express CD25 more densely than conventional T cells, and the transcription factor FoxP3 is associated with Treg cell function. Anti-CD25 mAb administration resulted in a 78% average reduction in circulating CD4^+^CD25^hi^ T cells (Figure 1A) and a 50% average reduction in CD4^+^CD25^+^FoxP3^+^ T cells by day 0 (Figure 1B).

### CD25 depletion does not significantly alter viral load during acute FIV infection

It has been hypothesized that Treg cells are either more frequently infected by latent lentivirus [9], [37] or are more efficient virion producers when infected as compared to conventional T cells [38], [39], [40], [41]. Treg cell depletion using the IL-2–toxin fusion protein denileukin diftitox prior to HIV-1 infection in humanized mice has been shown to reduce plasma viremia on days 4–10 p.i. [37]. It is therefore possible that reduced Treg cell levels at the onset of lentiviral infection could result in reduced viremia or provirus levels during acute infection. It is also possible that Treg cell depletion could reduce viral load due to heightened antiviral responses, or that Treg cell depletion could increase viral load due to elevated immune activation and viral replication.

**Figure 2:**
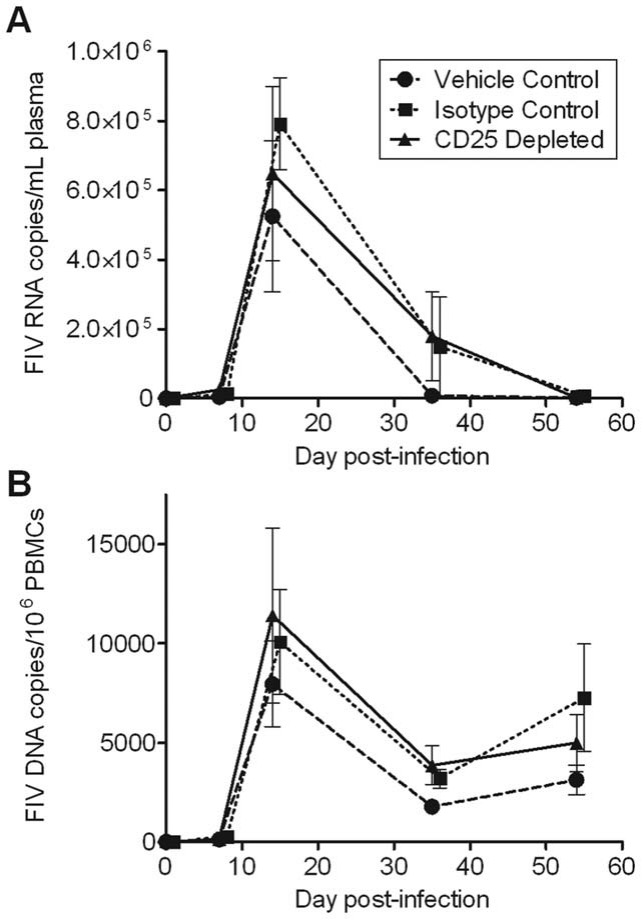
Anti-CD25 mAb treatment does not significantly alter FIV levels in the blood. Cats were infected with 2×10^5.2^ TCID_50_ units molecular clone FIV-C36 by intravaginal and intravenous routes on day 0. (A) To detect viremia in plasma samples, one-step quantitative real-time RT-PCR was performed on extracted RNA samples. FIV RNA copy number was quantified relative to an FIV C *gag* RNA standard curve. (B) Provirus in PBMCs was detected via quantitative real-time RT-PCR on extracted DNA samples. FIV copy number was quantified relative to an FIV C *gag* DNA standard curve, and then normalized relative to CCR5 copy number. Mean ± SEM is shown (n = 8/group).

**Figure 3:**
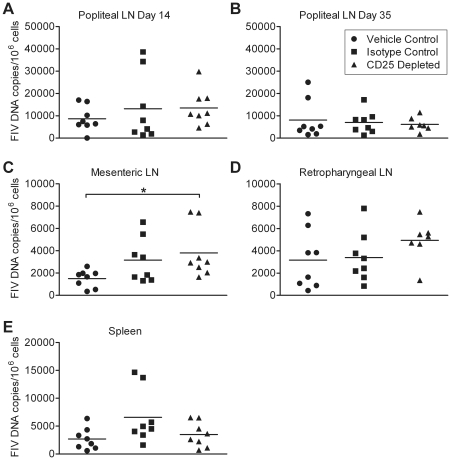
FIV proviral load in tissues is unaffected by anti-CD25 mAb treatment. (A–B) Provirus in popliteal lymph nodes was quantified on days 14 and 35 p.i. (C–E) Provirus in mesenteric and retropharyngeal lymph nodes and spleen was quantified on day 54 p.i. * indicates *p*<0.05.

We found that Treg cell depletion at the onset of FIV infection does not cause significant changes in viremia or proviral load. A trend of higher viremia and peripheral blood mononuclear cell (PBMC) proviral load was found in the CD25 depleted group as compared to the control groups on days 14 and 35 p.i. (Figure 2A and 2B). Proviral load was evaluated at intermediate time points in popliteal lymph nodes sampled on days 14 and 35 p.i.; however, no differences were observed among treatment groups (Figure 3A and 3B). In tissues evaluated terminally at day 54 p.i., proviral load was significantly higher in the mesenteric lymph nodes of CD25 depleted cats as compared to vehicle control cats, but not isotype control cats (Figure 3C). Similarly, proviral load in the retropharyngeal lymph nodes of CD25 depleted cats tended to be higher than in retropharyngeal lymph nodes of control cats (Figure 3D). However, no differences were observed in the spleen (Figure 3E). Both viremia and proviral levels reached set-point by day 54 p.i. in all cats (Figure 2 and 3). Our data indicate that anti-CD25 mAb treatment prior to FIV-C36 infection does not reduce and may in fact slightly augment viral load during acute infection.

### Lymphocyte dynamics and immune cell activation during acute FIV infection are not altered by anti-CD25 mAb treatment

**Figure 4:**
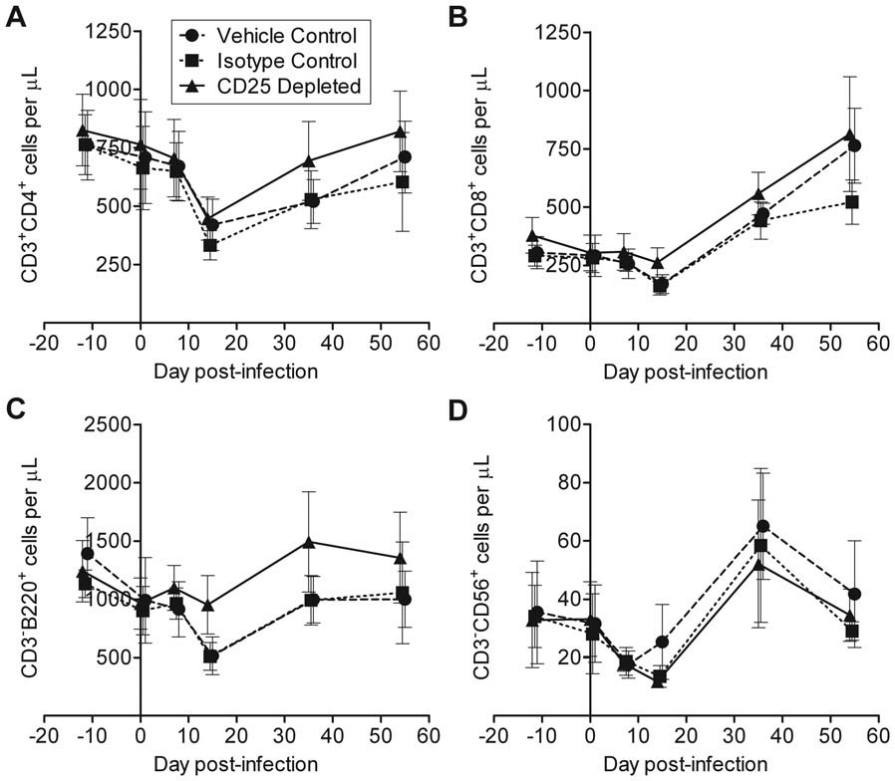
Peripheral lymphocyte dynamics during acute FIV-C36 infection are not altered by anti-CD25 mAb treatment. Absolute numbers of (A) CD3^+^CD4^+^, (B) CD3^+^CD8^+^, (C) CD3^−^B220^+^ (B cells), and (D) CD3^−^CD56^+^ (NK cells) lymphocytes in the periphery were quantified on days −12, 0, 7, 14, 35, and 54 p.i. based on percentages obtained by flow cytometry and absolute number of peripheral lymphocytes. Mean ± SEM is shown (n = 8/group).

Depletion of CD25^+^ Treg cells using anti-CD25 mAbs in acute infection models has raised concerns that there may be concomitant depletion of activated effector cells [42]. To avoid this, it was important to administer anti-CD25 mAb in a timeframe that allowed the majority of anti-CD25 mAb to bind to Treg cells or be cleared before effector cells were generated. We infected cats with FIV-C36 12 days after administration of anti-CD25 mAb, which allowed depletion of the majority of Treg cells before infection. Acute pathogenic FIV infection is characterized by a dramatic decline in peripheral CD4^+^ T cell counts with a slight decline in CD8^+^ T cells developing around or directly following the occurrence of peak viremia [32]. CD4^+^ T cell counts rebound to a degree but remain lower than pre-infection levels as they gradually decline throughout the course of the disease. Cytotoxic CD8^+^ T cells eventually expand to higher than baseline levels as they respond to infection [32], [43]. We observed characteristic CD4^+^ and CD8^+^ T cell dynamics during acute FIV-C36 infection (Figure 4A and 4B). In the control group cats, B cells were decreased on day 14 p.i., but cats in the CD25 depleted group did not experience a similar transient B cell decline (Figure 4C). Natural killer (NK) cells were decreased in all three groups on day 14 p.i., and an increase in NK cell absolute numbers was observed on day 35 p.i. (Figure 4D). No significant differences were observed in T cell, B cell, or NK cell dynamics between treatment groups in PBMC (Figure 4) or in lymphoid tissues (data not shown). There were also no differences in CD4∶CD8 T cell ratios in tissues, nor differences in cell density and cellularity in the lymph nodes between the groups (data not shown).

**Figure 5:**
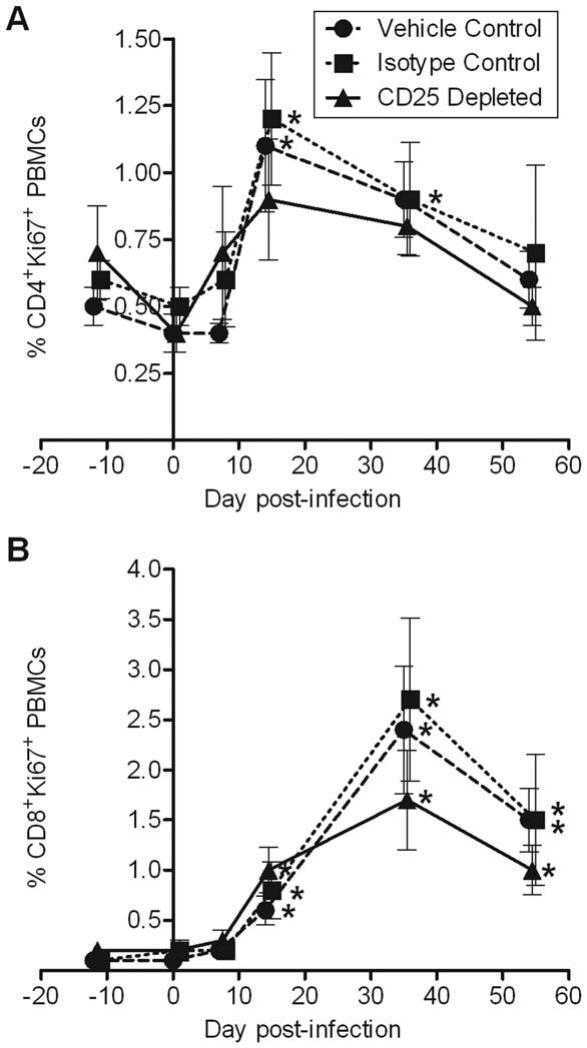
T cell proliferation due to FIV infection is not enhanced by anti-CD25 mAb treatment. Percent (A) CD4^+^ Ki67^+^ T cells and (B) CD8^+^ Ki67^+^ T cells in circulation were quantified by flow cytometry on days −12, 0, 7, 14, 35, and 54 p.i. (n = 8/group). Mean ± SEM is shown. Significance is relative to day 0 values for each group. No significant differences were found between treatment groups. * indicates *p*<0.05.

Treg cells have been shown to suppress lentiviral-specific T cell proliferation ex vivo [16], [44]. We asked whether Treg cell depletion in vivo could augment T cell proliferation after FIV infection. T cell proliferation in the periphery was determined via Ki67 expression. CD4^+^ T cell proliferation was significantly increased in control groups on day 14 p.i., corresponding with the CD4^+^ T cell nadir and peak viremia (Figure 5A). CD8^+^ T cell proliferation was significantly increased at days 14, 35, and 54 and was maximal at day 35 p.i. in all three treatment groups (Figure 5B). Peripheral Ki67^+^ B cell and Ki67^+^ NK cell frequencies did not change significantly after FIV infection and were not significantly different between the groups (data not shown). Ki67 expression by lymphocyte subsets in tissues was not significantly different between the groups on days 14, 35, or 54 p.i. (data not shown).

### Cytokine-expressing CD8^+^ T cell levels are lower after anti-CD25 mAb treatment on day 35 post-FIV infection

**Figure 6:**
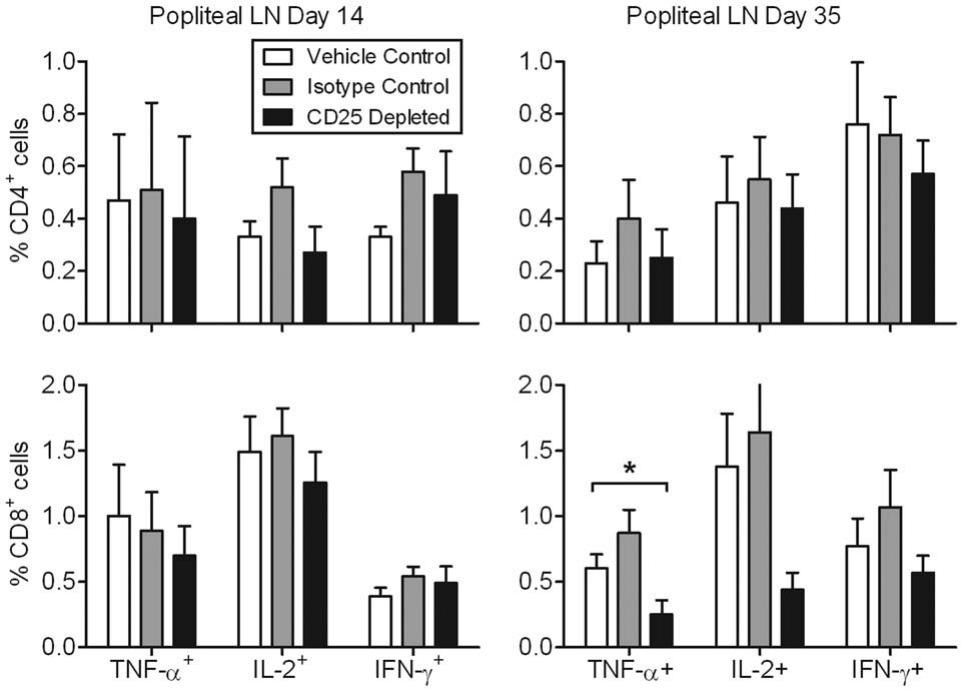
Cytokine-expressing CD8^+^ T cell levels are lower after anti-CD25 mAb treatment on day 35 post-FIV infection. Popliteal lymph node cells were incubated with monensin for 6 hours prior to quantification of cytokine expressing cells. Percent TNF-α-, IL-2-, and IFN-γ-expressing cells in CD4^+^ and CD8^+^ T cell populations were quantified by flow cytometry (n = 8/group). Mean ± SEM is shown. * indicates *p*<0.05.

Intracellular expression of cytokines TNF-α, IL-2, and IFN-γ was determined in popliteal lymph node CD4^+^ and CD8^+^ T cells during acute FIV infection in controland CD25 depleted cats. No significant differences in cytokine expression by T cells were found between the treatment groups at day 14 p.i. (Figure 6). In contrast, CD8^+^TNF-α^+^ cell levels were significantly lower in the CD25 depleted group as compared to the vehicle control group at day 35 p.i., and there was a trend of lower IL-2 and IFN-γ expression by CD8^+^ T cells in the CD25 depleted group as well. Cytokine expression by CD4^+^ T cells at day 35 p.i. was not significantly different between the groups (Figure 6). By day 54 p.i. no differences were observed in T cell cytokine expression between treatment groups in mesenteric lymph node, retropharyngeal lymph node, and spleen (data not shown).

### Cellular and humoral anti-FIV specific responses are slightly depressed in anti-CD25 mAb treated cats

**Figure 7:**
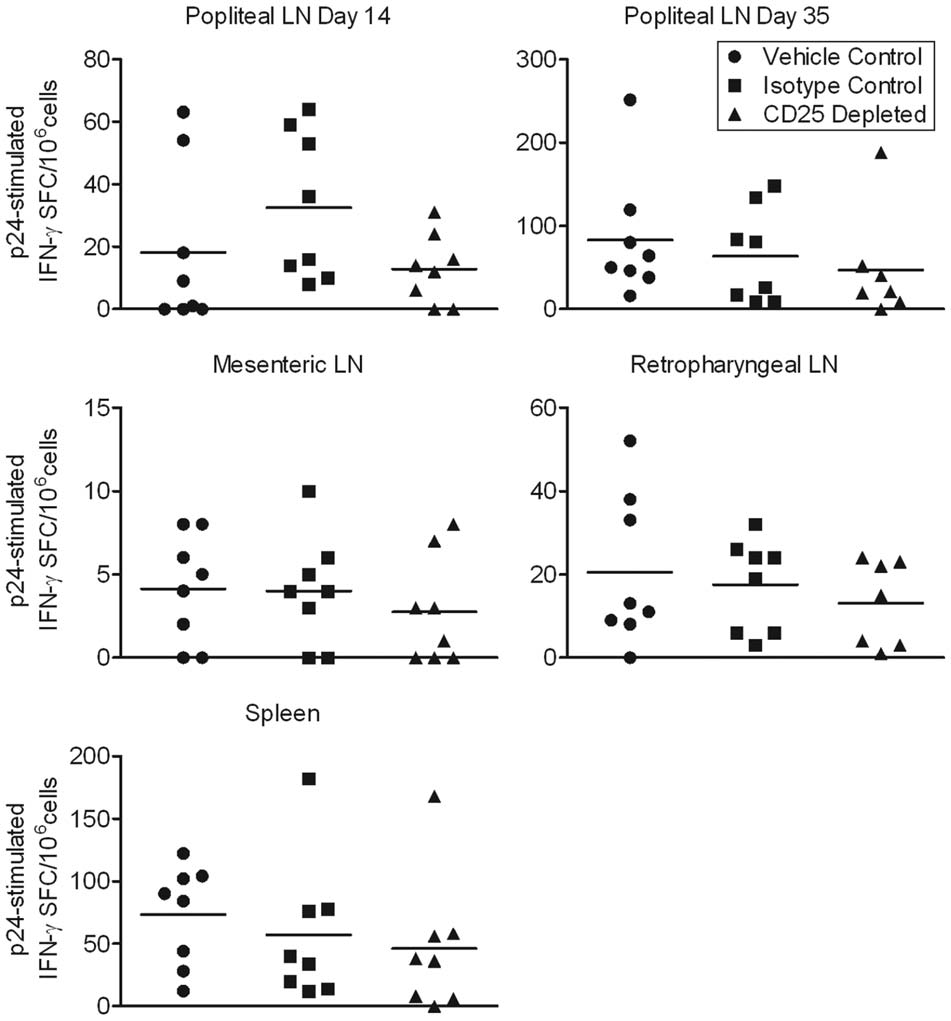
FIV-specific IFN-γ responses during acute infection are not altered by anti-CD25 mAb treatment. Popliteal lymph node cells from day 14 and 35 p.i. and mesenteric and retropharyngeal lymph node cells and spleen cells from day 54 p.i. were incubated for 48 hours with 100 µg/mL FIV p24 peptides or 1% DMSO/media as a background control. IFN-γ spot forming cells (SFCs) per million cells from CD25 depleted, isotype control, and vehicle control treated groups in response to FIV p24 minus background were determined by ELISpot (n = 8/group).

We have previously shown that in vivo Treg cell depletion in cats chronically infected with FIV unmasks existing FIV-specific IFN-γ secreting cells [18]. We asked whether CD25 depletion before FIV infection would allow a more robust induction of FIV-specific immune responses. Lymphocytes were stimulated with FIV p24 peptides in an IFN-γ ELISpot assay. No significant differences were found between the groups; however, there was a trend of fewer FIV-specific IFN-γ secreting cells in the CD25 depleted group as compared to the control groups in tissues analyzed from days 14, 35, and 54 p.i. (Figure 7).

**Figure 8:**
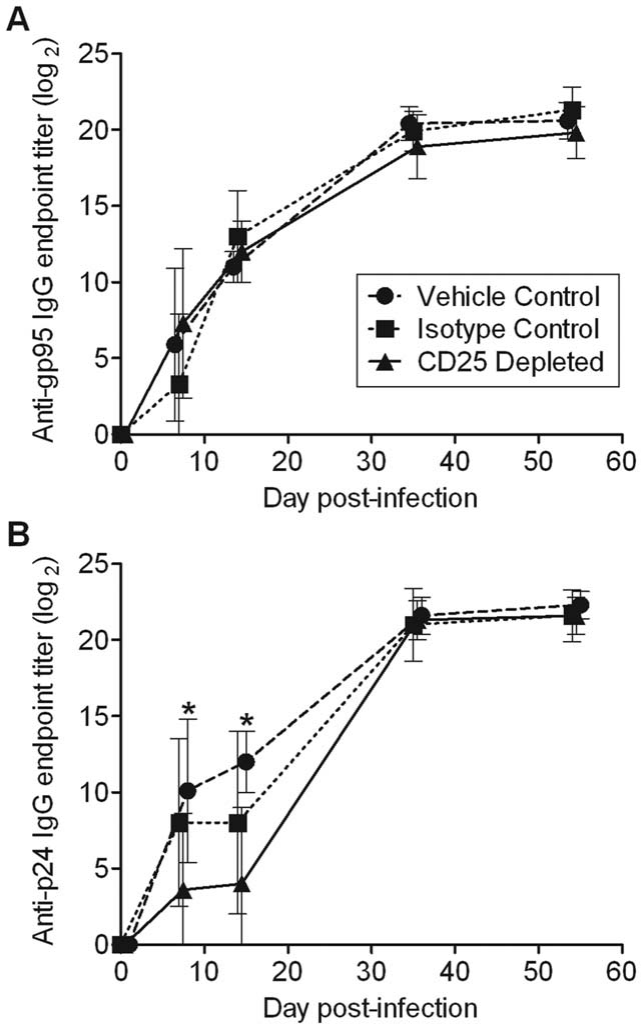
Anti-FIV p24, but not anti-FIV gp95, IgG titers are significantly lower after anti-CD25 mAb treatment during acute FIV infection. (A) Anti-FIV gp95 and (B) anti-FIV p24 IgG titers were quantified in serum samples on days 0, 7, 14, 35, and 54 post-FIV infection. Positive antibody titers were calculated based on a 3-fold higher optical density than day 0 pre-inoculation controls. Mean ± SEM is shown. Statistical significance was determined between treatment groups for each time point (n = 8/group). * indicates *p*<0.05.

Neutralizing antibodies have been shown to play a role in controlling FIV replication. Anti-FIV gp95 IgG titers were not significantly different between the groups during acute FIV infection (Figure 8A); however, anti-FIV p24 IgG titers were significantly higher in the vehicle control group as compared to the CD25 depleted group on days 7 and 14 p.i. (Figure 8B). These data may explain why viral load was slightly higher in the CD25 depleted group at certain time points during acute infection.

### Kinetics and characterization of Treg cell induction during acute FIV infection

**Figure 9:**
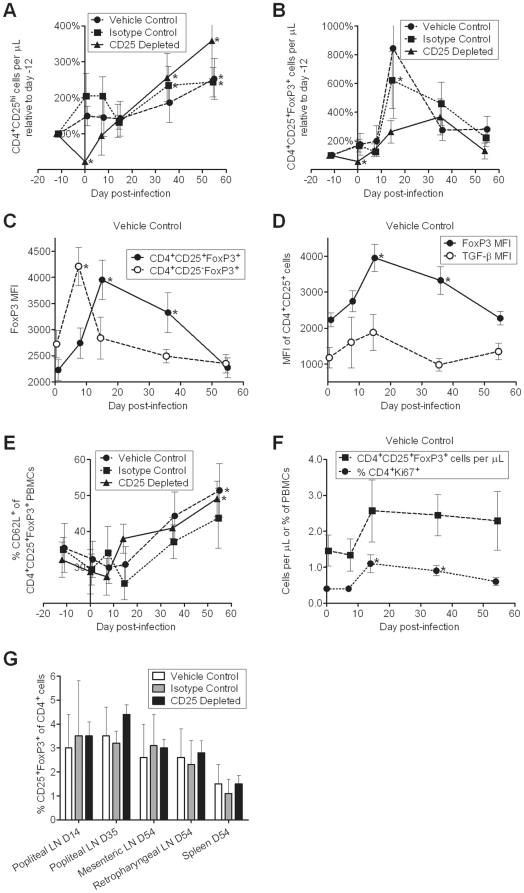
Peripheral Treg cells are induced during acute FIV infection. (A) CD4^+^CD25^hi^ percentages within the PBMC population were determined by flow cytometry on days −12, 0, 7, 14, 35, and 54 post-FIV infection. Percentages were multiplied by lymphocyte absolute number to quantify absolute numbers of CD4^+^CD25^hi^ cells. Absolute numbers were then expressed as a percentage of day −12 values and averaged. (B) CD4^+^CD25^+^FoxP3^+^ percentages within the PBMC population were determined by flow cytometry, then absolute numbers were expressed as a percentage of day −12 values and averaged. (A, B) Significance was determined relative to day −12 values. * indicates *p*<0.05. (C) FoxP3 mean fluorescence intensity (MFI) within CD4^+^CD25^+^FoxP3^+^ and CD4^+^CD25^−^FoxP3^+^ PBMCs from vehicle control-treated cats was determined by flow cytometry. Levels and kinetics did not significantly differ between treatment groups. (D) FoxP3 and TGF-β MFI within CD4^+^CD25^+^ PBMCs from vehicle control-treated cats was determined by flow cytometry. Levels and kinetics did not significantly differ between treatment groups. (E) Percent of CD4^+^CD25^+^FoxP3^+^ PBMCs expressing CD62L was determined by flow cytometry. (F) Percent CD4^+^Ki67^+^ PBMCs from vehicle control-treated cats was determined by flow cytometry and is compared to CD4^+^CD25^+^FoxP3^+^ PBMC absolute number. Levels and kinetics did not significantly differ between treatment groups. (C–F) Significance was determined relative to day 0 values. * indicates *p*<0.05. (G) Percent of CD4^+^ T cells expressing CD25 and FoxP3 in lymphoid tissues was determined by flow cytometry on days 14, 35, or 54 post-FIV infection. (A–G) Mean ± SEM is shown.

Throughout the study, we tracked Treg cell levels and properties. We found that peripheral Treg cell frequency increases substantially during acute FIV infection. Peripheral CD4^+^CD25^hi^ cells were slightly reduced at day 14 p.i. in the isotype control and the vehicle control groups (Figure 9A). This corresponded with peak viremia and depletion of total CD4^+^ T cells as a result of pathogenic FIV infection (Figure 2 and 4A). In all groups CD4^+^CD25^hi^ cells exceeded baseline levels by day 35 p.i. (Figure 9A). In the anti-CD25 mAb treated group, peripheral CD4^+^CD25^+^FoxP3^+^ cells exceeded baseline levels by day 14 p.i. (Figure 9B).

CD4^+^CD25^+^FoxP3^+^ cells rebounded more quickly and to a higher degree after CD25 depletion in acutely infected cats as compared to FIV naïve or chronically infected FIV^+^ cats (Figure 1B) [18], [22]. Control group cats also exhibited increased absolute numbers of CD4^+^CD25^+^FoxP3^+^ cells by day 14 p.i. (Figure 9B). This effect is presumably due to Treg cell conversion and/or proliferation in response to FIV infection. We found that FoxP3 protein expression increased in CD4^+^CD25^−^FoxP3^+^ cells by day 7 p.i., and this was followed by a decrease in FoxP3 expression by CD4^+^CD25^−^ cells and an increase in FoxP3 expression by CD4^+^CD25^+^cells at day 14 p.i. (Figure 9C). This suggests that CD4^+^CD25^−^FoxP3^+^ cells were converting to CD4^+^CD25^+^FoxP3^+^ cells between days 7 and 14 p.i. In addition, TGF-β protein expression increased in CD4^+^CD25^+^ cells at the same time FoxP3 protein expression increased (Figure 9D). TGF-β has been associated with one mechanism of Treg cell suppression in cats [45]. CD62L^+^ cell frequency also increased within the CD4^+^CD25^+^FoxP3^+^ cell population throughout the study (Figure 9E). The early activation marker CD62L has been shown to distinguish Treg cells from T effector cells in mice [46]. CD4^+^Ki67^+^ cell kinetics in the periphery mirror the increase in absolute number of CD4^+^CD25^+^FoxP3^+^ cells (Figure 9F). This suggests that Treg cell proliferation may contribute to the increase in peripheral Treg cell absolute number during acute FIV infection. These data indicate increased peripheral Treg cell presence in all cats during acute FIV-C36 infection regardless of treatment.

CD4^+^CD25^+^FoxP3^+^ cell levels were not reduced in popliteal lymph nodes at day 14 or day 35 p.i. in the CD25 depleted group as compared to the control groups (Figure 9G). We were not able to determine changes in Treg cell levels in tissues after FIV infection, as lymph nodes were not biopsied prior to infection.

## Discussion

Regulatory T cells have been shown to suppress function of CD4^+^ and CD8^+^ effector T cells, natural killer cells, and dendritic cells. During chronic FIV, SIV or HIV-1 infection it is clear that Treg cells suppress antiviral responses in vitro [10], [12], [13], [14]. We have shown that this suppression is also exerted in vivo during chronic FIV infection [18]. Whether the net effect of suppressing chronic immune activation is beneficial likely depends on the immunologic and virologic set-point of any given individual. The present study sought to determine whether Treg cell depletion at the time of FIV infection might allow a more robust anti-viral immune response that would result in an improved immunologic set-point and a decreased virologic set-point. Our results show this is not the case. Treatment with anti-CD25 antibody effectively depleted Treg cells as measured by the frequency of CD4^+^CD25^hi^ cells or CD4^+^CD25^+^FoxP3^+^ cells (Figure 1). Nevertheless, antigen specific CD8^+^ T cell responses were not improved over control animals and neither were antiviral antibody titers. Similarly, viral burden, whether measured as plasma viremia or proviral copy number in blood or tissues, was the same or greater in Treg cell depleted cats. Thus we conclude that Treg cells present at the time of infection do not alter the immune response against FIV. In addition, our data indicate that Treg cells present at the time of FIV infection are not major reservoirs of virus during the first few days after infection.

Depletion of Treg cells at the time of antigenic stimulation has resulted in variable outcomes. Several studies have shown that Treg cell depletion immediately prior to or at the time of vaccination results in increased antigen-specific responses to the vaccine [47], [48], [49], [50]. Results from similar studies involving Treg cell depletion at the time of challenge with an infectious agent have been variable. For example, Treg cell depletion did not improve the immune response in mice challenged with *Plasmodium yoelii*, *Trypanosoma cruzi* or *Psuedomonas aruginosa* [51], [52], [53]. Even though Treg cell depleted mice challenged with rotavirus had improved antigen-specific CD4^+^ and CD8^+^ T cell responses, the clinical outcome was not improved [54]. The difference between immune induction with vaccines versus infectious agents under conditions of Treg cell depletion may be related to the strength of activation signals to effector versus regulatory T cells. It has been shown that production of certain cytokines or ligation of certain toll-like receptors during infection can either render conventional T cells refractory to Treg cell suppression or temporarily disrupt Treg cell suppressive function [55]. It seems likely that infectious agents provide a greater quantity of antigen, DC activation, and IL-2 production as compared to the limited antigen available by vaccination. This would result in T effectors that are more likely to be refractory to Treg cell suppression in the infection scenario. The importance of the strength of T-cell receptor signals has been clearly demonstrated for HIV-specific responses by Antons et al. [56]. FIV-C36 acute infection causes massive immune activation, and we did not observe changes in effector cell activation after Treg cell depletion and infection. It is likely that during acute FIV infection effector cells are resistant to any concurrent immunosuppressive effects mediated by Treg cells.

One important caveat regarding the present study and those mentioned previously is that anti-CD25 mAb was used for Treg cell depletion. This approach is limited in its specificity and efficacy. CD25 is expressed by many cell types, including recently activated T effector cells, thereby limiting the utility of anti-CD25 treatment to the time frame prior to effector T cell activation. Furthermore, not all CD25^+^ Treg cells are depleted by the mAb nor do all Treg cells express CD25. The development of mice that are transgenic for co-expression of FoxP3 and diphtheria toxin receptor allows selective depletion of nearly all FoxP3 expressing cells [57]. Zelinskyy et al. exploited this system in conjunction with Friend retrovirus infection and showed continuous FoxP3^+^ cell depletion resulted in increased CD8^+^ T cell responses and lower viral burden [58]. These results suggest the acute antiviral response might be improved by Treg cell depletion but the depletion must be sustained during the time when Treg cells are simultaneously induced with T effector cells. Obviously such an approach is not possible in humans or outbred models such as the cat, nor is FoxP3 exclusively expressed in Treg cells of either of these species [59], [60]. Investigation of clinically feasible Treg cell manipulation for human patients is limited to the currently available strategies that target CD25, including the IL-2–toxin fusion protein denileukin diftitox and anti-CD25 mAbs. Treg cell depletion may be more effective in the presence of low antigen levels as is the case with most vaccines. Under conditions of low antigen the balance between effector responses versus Treg cell suppression is more likely to be tipped toward Treg cell suppression. This could mean that Treg cell depletion before vaccination would boost immunity. In support of this hypothesis, it has been shown that HIV-1-specific Treg cells induced after therapeutic DC vaccination in HIV-1-infected patients significantly inhibit development of polyfunctional CD8^+^ T cell responses [61]. It remains to be determined whether Treg cell depletion prior to therapeutic HIV-1 vaccination would be sufficient to reduce immunosuppression in a clinically significant manner.

Despite Treg cell depletion prior to FIV infection, the kinetics of Treg cell induction associated with FIV were not altered. The absolute number of CD4^+^CD25^+^FoxP3^+^ cells was increased at day 14 p.i. and remained elevated through day 35 p.i., reaching a plateau or declining by day 54 p.i. We hypothesize that elevation in peripheral CD4^+^CD25^+^FoxP3^+^ cell numbers was due to new expression of CD25 by cells that were CD4^+^CD25^−^FoxP3^+^ at the time of FIV infection. Our data show that FoxP3 protein expression increased in CD4^+^CD25^−^FoxP3^+^ cells by day 7 p.i., and this was followed by a decrease in FoxP3 expression by CD4^+^CD25^−^ cells and an increase in FoxP3 expression by CD4^+^CD25^+^cells at day 14 p.i. (Figure 9C). This suggests that FoxP3 upregulation was followed by CD25 expression and a transition of cells to the phenotype CD4^+^CD25^+^ known to harbor activated, suppressive Treg cells in the cat [22]. This idea is supported by adoptive transfer studies in the mouse showing that de novo Treg cell generation during acute malaria infection was not due to proliferation of CD25^+^FoxP3^+^ cells or differentiation of CD25^−^FoxP3^−^ cells but rather was the result of proliferation of CD25^−^FoxP3^+^ cells that simultaneously began expressing CD25 [62]. These studies demonstrated suppressive function in the CD4^+^CD25^−^FoxP3^+^ population although the lack of CD25 expression might be an artifact of in vivo treatment with the anti-CD25 mAb 7D4, that is reported to induce shedding of CD25 [62]. It is not possible to functionally assess the CD25^−^FoxP3^+^ population in cats since FoxP3 cannot be detected in viable cells. However, it has been repeatedly shown that the suppressive feline Treg cell population resides within the CD4^+^CD25^+^ phenotype [9], [10], [22]. Taken together, the present study and previous reports support the idea that feline Treg cells are defined by FoxP3, that activated Treg cells coexpress FoxP3 and CD25, and that the CD4^+^CD25^−^FoxP3^+^ population represents naïve Treg cells with proliferative potential. This remains to be confirmed in future studies.

A question that remains is whether infection with FIV, SIV, or HIV-1 preferentially drives Treg cell production and/or activation during the acute phase of infection. It may be that viral proteins directly contribute to the activation of Treg cells. Nilsson et al. demonstrated increased FoxP3 expression and decreased apoptosis in Treg cells cultured with HIV-1 gp120 and this is mediated through CD4. The primary receptor used by FIV is CD134 (OX40), not CD4; however, OX40 agonists have been shown to induce the proliferation and accumulation of Treg cells in mice [63]. Another possible factor contributing to Treg cell induction could be the immune response associated with pathogenic FIV, SIV, or HIV-1 infection. Partially activated dendritic cells are associated with HIV-1 infection and Treg cell induction [64], [65]. In the Friend retrovirus model it has been shown that expansion of the Treg cell population is dependent on the presence of CD4^+^ and CD8^+^ effector T cells and is independent of the level of viremia [58], [66], [67]. Thus, robust T effector cell induction may provide the cytokine milieu needed for Treg cell expansion during FIV/SIV/HIV infections. Several studies have demonstrated increased Treg cell∶T effector cell ratios during SIV and HIV-1 infection [19], [68], [69]. During acute SIV infection, Treg cell induction correlated with Ki67 expression on lymphocytes [19], similar to what we observed in this study (Figure 9F). Determining the mechanism behind the Treg cell response may be important with regard to vaccine development.

In conclusion, we report here that Treg cells present before infection do not play a major regulatory role during acute FIV pathogenesis. However, we found that CD4^+^CD25^+^FoxP3^+^ Treg cells are rapidly induced in the periphery during acute FIV infection. Because Treg cells have been shown to be activated and play a role in antiviral immunosuppression during chronic FIV infection, we hypothesize that Treg cell induction during acute FIV infection leads to the development of an activated Treg cell population that plays a major role in suppression of antiviral responses during chronic infection [10], [18], [70]. Additional studies to determine whether Treg cell depletion prior to therapeutic vaccination during chronic lentiviral infection can boost immunity should be performed.
